# spicyR: spatial analysis of *in situ* cytometry data in R

**DOI:** 10.1093/bioinformatics/btac268

**Published:** 2022-04-19

**Authors:** Nicolas P Canete, Sourish S Iyengar, John T Ormerod, Heeva Baharlou, Andrew N Harman, Ellis Patrick

**Affiliations:** The Westmead Institute for Medical Research, University of Sydney, Westmead, NSW, Australia; Sydney Medical School, University of Sydney, Sydney, NSW, Australia; School of Mathematics and Statistics, University of Sydney, Sydney, NSW, Australia; School of Mathematics and Statistics, University of Sydney, Sydney, NSW, Australia; The Westmead Institute for Medical Research, University of Sydney, Westmead, NSW, Australia; Sydney Medical School, University of Sydney, Sydney, NSW, Australia; The Westmead Institute for Medical Research, University of Sydney, Westmead, NSW, Australia; Sydney Medical School, University of Sydney, Sydney, NSW, Australia; The Westmead Institute for Medical Research, University of Sydney, Westmead, NSW, Australia; School of Mathematics and Statistics, University of Sydney, Sydney, NSW, Australia

## Abstract

**Motivation:**

High parameter histological techniques have allowed for the identification of a variety of distinct cell types within an image, providing a comprehensive overview of the tissue environment. This allows the complex cellular architecture and environment of diseased tissue to be explored. While spatial analysis techniques have revealed how cell–cell interactions are important within the disease pathology, there remains a gap in exploring changes in these interactions within the disease process. Specifically, there are currently few established methods for performing inference on cell-type co-localization changes across images, hindering an understanding of how cellular environments change with a disease pathology.

**Results:**

We have developed the spicyR R package to perform inference on changes in the spatial co-localization of types across groups of images. Application to simulated data demonstrates a high sensitivity and specificity. We the utility of spicyR by applying it to a type 1 diabetes imaging mass cytometry dataset, revealing changes in cellular associations that were relevant to the disease progression. Ultimately, spicyR allows changes in cellular environments to be explored under different pathologies or disease states.

**Availability and implementation:**

R package is freely available at http://bioconductor.org/packages/release/bioc/html/spicyR.html and shiny app implementation at http://shiny.maths.usyd.edu.au/spicyR/.

**Supplementary information:**

Supplementary data are available at *Bioinformatics* online.

## 1 Introduction

Identifying changes in the spatial distribution of cells is vital for understanding the cellular processes that are present in diseased tissue. Multiplexed histological techniques have allowed the complex cellular architecture and environment of diseased tissue to be explored by enabling the simultaneous profiling of multiple cell types. Fluorescence-based methods, including co-detection by imaging (CODEX) (Goltsev *et al.*, 2018), cyclic immunofluorescence (Lin *et al.*, 2015) and iterative indirect immunofluorescence imaging (4i) (Gut *et al.*, 2018), as well as mass cytometry imaging techniques, including imaging mass cytometry (IMC) (Giesen *et al.*, 2014) and multiplexed ion beam imaging by time of flight (Angelo *et al.*, 2014) allow up to 40 protein markers to be visualized with single-cell resolution. Additionally, the development of spatial-based transcriptomic techniques, such as High-Definition Spatial Transcriptomics (Vickovic *et al.*, 2019) and sequential fluorescence *in situ* hybridization (Lubeck *et al.*, 2014), allow tens of thousands of transcripts to be spatially resolved within an image at single-cell resolution. This large increase in the scale and dimensionality of the images being acquired has necessitated the development of analysis techniques capable of interrogating such complex data.

Established image analysis techniques have enabled the investigation of cell–cell interactions and cell migration within an image, facilitating the interrogation of high parameter imaging data in a single-cell manner. Standard pipelines start by identifying cells through single-cell segmentation followed by cell-type classification by clustering or manually gating marker expression (Baharlou *et al.*, 2019; Carpenter *et al.*, 2006; Sommer *et al.*, 2011; van Valen *et al.*, 2016). From here, a differential analysis of cell composition or marker expression within the image dataset can be performed to identify high-level associations with a phenotype of interest. The spatial dimension afforded by imaging can furthermore allow the spatial context of these cells to be quantified in multiple ways. One way to interrogate the spatial organization of cells is to quantify the spatial attraction or avoidance between pairs of cell types. This often involves counting an association measure between cell types. Counting the number of touching cells of a pair of cell types provides a measure of spatial association (Schapiro *et al.*, 2017). Randomizing the labels of cells can then be used to identify how significant a pairwise interaction is within an image, as seen in an application to type 1 diabetes images (Damond *et al.*, 2019). This approach can be extended by calculating distances between cells and tabulating their nearest neighbours as used by Keren *et al.* to assess the spatial interactions involved in triple-negative breast cancer pathology (Keren *et al.*, 2018). Ripley’s K-function (Baddeley *et al.*, 2015; Ripley, 1976) can be used to assess how co-localization between cell types vary with distance by modelling cells within an image as a point process. Further to this, identification of spatial communities (Jackson *et al.*, 2020) has been performed through graph-based techniques, associating the spatial distribution of groups of cell types to disease outcomes. Finally, techniques, such as spatial variance component analysis (Arnol *et al.*, 2019), have been developed to identify the sources of variation of gene or protein markers attributed to cell–cell interactions. Overall, such techniques can identify spatial structure within high parameter images. This could then be attributed to the pathology of the disease states being studied.

A key gap that remains in the analysis of high parameter images is the identification of differential cell-type co-localization across groups—i.e. changes in the extent of pairwise cell-type co-localization across these groups. These groups could be a clinical outcome, such as disease stage or response to treatment or come from a perturbed experiment. Present strategies involve comparing association measures across groups. Damond et al. (2019) compares the number of each pairwise interaction using a Mann–Whitney’s *U*-test to show changes in cell-type co-localizations across different disease stages. Färkkilä *et al.* (2020) compares the *Z*-scores of the pairwise cell interaction obtained from bootstrapping across ovarian cancer clinical outcomes to identify changes in cell distribution. While such approaches are appropriate, they do not allow for the modelling of multiple images from multiple subjects. Additionally, these strategies ignore the variation observed in the quantification of cell-type co-localization.

In this manuscript, we present an R package ‘SPatial analysis of In situ CYtometry data in R’ (spicyR) and a corresponding web application to facilitate inference on changes in spatial co-localizations between cell types. spicyR aims to provide an easy-to-use approach to identify differential cell-type co-localization with respect to a disease or treatment and has the capacity to model information from multiple images per subject or account for images having a substantial difference in cell number. We demonstrate the performance of spicyR through simulation and apply the package to the diabetes imaging data presented in Damond *et al.* (2019), revealing changes in cellular co-localization with type 1 diabetes progression.

## 2 Materials and methods

**Fig. 1.**
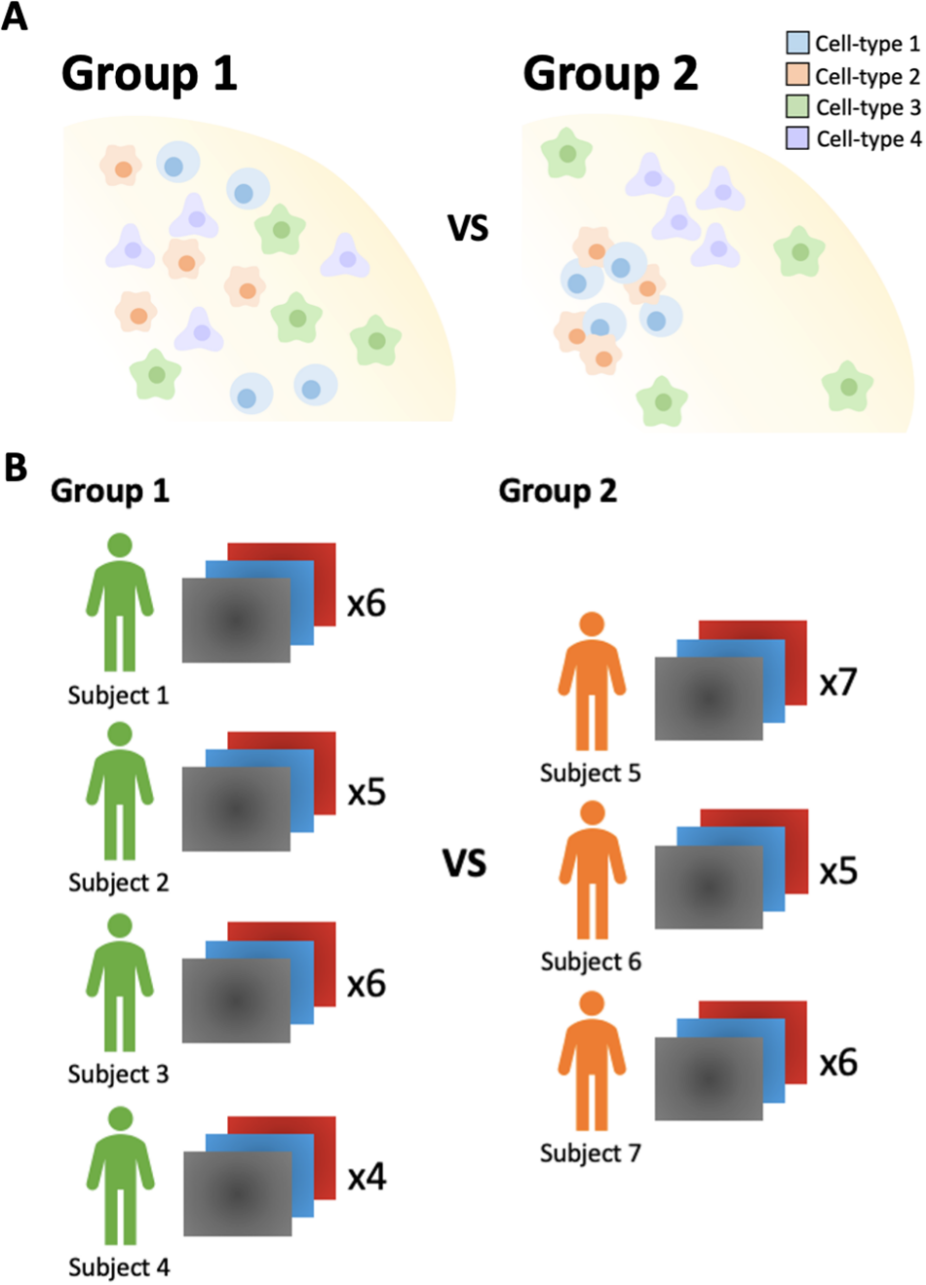
Schematic of the experimental motivation of spicyR. (**A**) Analytical question. From the images obtained, spicyR aims to identify differences in cell-type co-localizations between the two groups. (**B**) Example experimental setup. Here, we have four subjects from Group 1, and three donors from Group 2, each with a different number of high parameter images. When applying spicyR, the number of subjects per group does not have to be equal, the number of images per subject does not have to be equal, there can be one image for subject. Further to this, the number of groups can be larger than two or alternatively continuous outcome

Our R package, spicyR, provides the framework for performing inference on the changes in spatial co-localizations between pairs of cell types, which can be associated with a discrete or continuous outcome (Fig. 1A). As described below, spicyR consists of three primary steps: (i) summarizing the degree of spatial co-localization between pairs of cell types for each image; (ii) modelling the variability in the co-localization summary statistics as a function of cell counts and (iii) testing for changes in co-localization associated with a response variable. The significance of this change is assessed using a linear model, or a mixed-effects model if there are multiple images belonging to a subject (Fig. 1B). The R package is available on Bioconductor (http://bioconductor.org/packages/release/bioc/html/spicyR.html), and is also implemented as an interactive shiny application (http://shiny.maths.usyd.edu.au/spicyR/).

### 2.1 Construction of the *L* curve and co-localization score

Following single-cell segmentation and classification, images are modelled as a ‘marked point process model’, in which each cell is represented as a point in a 2D plane. Spatial co-localization between two cell types within an image can be quantified with a *K*-function (Ripley, 1976),

(1) $${\hat{K}}_{ij}\left(r\right)=\frac{\left|W\right|}{n_{i}n_{j}}\sum_{n_{i}}\sum_{n_{j}}1\left\{d_{ij}\leq r\right\}e_{ij}\left(r\right),$$

where $\hat{K_{ij}}\left(r\right)$ summarized the degree of co-localization of cell type *j* with cell type *i*, $n_{i}$ and $n_{j}$ are the number of cells of type *i* and *j*, $\left|W\right|$ is the image area, $d_{ij}$ is the distance between two cells and $e_{ij}\left(r\right)$ is an edge correcting factor. The *K*-function can be interpreted as the average number of cells of type *j* within a distance r away from each cell of type *i*.

**Fig. 2.**
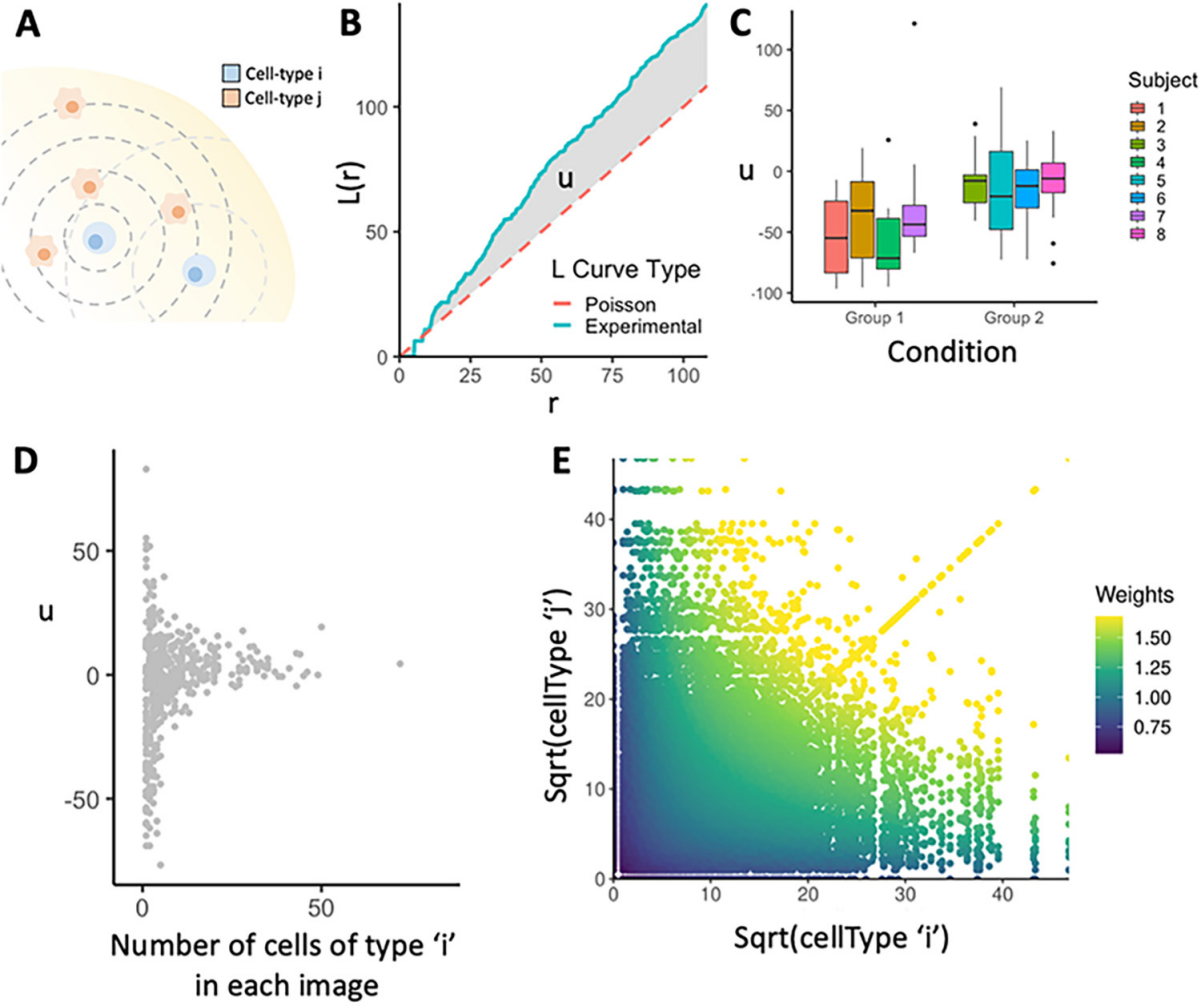
Summary of the spicyR framework. (**A**) Representation of two cell types in a 2D plane, which can be represented as points. Co-localization of cell type *j* around cell type *i* can be modelled by using Ripley’s *K*-function, which summarizes a scaled average of the number of cell type *j* around cell type *I* for a range of radii, *r* (dashed lines). (**B**) Example of an *L* curve generated from an experiment (solid line) compared against a *L* curve (dashed line) expected if cell type *i* and *j* were distributed independent of each other. The shaded area represents the *u* statistic used to assess whether co-localization (positive value) or avoidance (negative value) is occurring. (**C**) A boxplot representation of the *u* statistics used to compare pairwise co-localization across different groups. (**D**) Plot of the *u* statistic versus the number of cells of type i. As cell count is decreased, the variance of the AUC statistic increases. A GAM is used to model the square of the statistic as a 2D function of the counts of cell types ‘I’ and ‘j’, and these are used as weights in the linear models used. (**E**) Plot representing the weighting regime when applied to the dataset from Schürch *et al.* (2020). The value of the weights increases as the pairwise cell counts increases

The *L*-function or *L* curve is a variance stabilized *K*-function given by the equation

(2) $${\hat{L}}_{ij}\left(r\right)=\sqrt{\frac{{\hat{K}}_{ij}\left(r\right)}{r}}$$

(Ripley, 1976). Figure 2B shows an example observed *L* curve compared to the expected *L* curve obtained from points distributed randomly in a Poisson distribution. Curves above the Poisson line can be interpreted as showing greater attraction when compared to a random distribution, and curves below the Poisson line can be interpreted as avoidance of the two cell types.

To reduce these summary functions into a single co-localization score u, we take an area between curve measurement (Fig. 2B), given by the equation

(3) $$u=\sum_{{r^{'}=r}_{\min}}^{r_{\max}}{\hat{L}}_{ij,\mathrm{Experimental}}\left(r^{'}\right)-{\hat{L}}_{ij,\mathrm{Poisson}}\left(r^{'}\right),$$

where the sum is taken over a discrete range of *r* between *r*_min_ and *r*_max_ (e.g. *r’* *=* 10, 20, …, *r*_max_). A special case of Equation (3) that is explored below is when there is only one value to sum over (i.e. *r*_min_ *=* *r*_max_).Values of *u* > 0 represent greater attraction between cells when compared to random, while values <0 represent avoidance when compared to random.

### 2.2 Weighting scheme used to account for varying cell counts

The relationship between cell count and variability of *u* is modelled with a shape constrained generalized additive model (GAM) fitted with SCAM (Pya and Wood, 2015). We fit a GAM with a monotone decreasing constraint to the square of *u* with the counts of both cell types i and j as explanatory variables. The inverse of this fitted curve is used as a weighting scheme for each measurement in the model described in the following section—i.e. lower weights are applied to images with lower cell counts. These weights can be constructed per cell-type pair or using the scores from all cell types. In all the analyses in this manuscript the weights are constructed using all cell types concurrently.

### 2.3 Hypothesis testing with a linear model

To assess changes in cell-type co-localization between groups, we implement either a linear model, or a linear mixed-effects model with random intercepts using the lme4 package (Bates *et al.*, 2015). Here, $u_{ij}$ is the co-localization score for subject *i* and image *j*, the treatment group or condition ($x_{i}$) is modelled as a fixed effect with coefficient *β* and if a subject has multiple images the intercept ($\alpha_{i}$) is modelled as a random effect. Other covariates are included as *W* with coefficients Γ.

*Linear model:*
 

(4) $$u_{i}=\alpha +\beta x_{i}+\Gamma W.$$

*Linear mixed-effects model:*
 

(5) $$u_{ij}=\alpha_{i}+\beta x_{i}+\Gamma W.$$

For the mixed-effects model, *P*-values are calculated with Satterthwaite’s approximations using the lmertest package (Kuznetsova *et al.*, 2017).

### 2.4 Simulations to assess the performance of spicyR

To examine the performance of spicyR, simulations were generated using the spatstat package (Baddeley and Turner, 2005), as summarized below and in Supplementary Figure S2. There are a few key parameters in the simulation with their default values listed as follows.

- The size of each image: 1000×1000 units.
- The number of subjects: 40, split into 2 groups of 20 each.
- The number of images per subject: three, each containing two cell types.
- The number of simulations: 500 without change (*null* simulation) and 500 with change in co-localization between groups (*difference* simulations).
- A range for the number of cells of each type per image: *cell count* =20, 40, …, 380, 400.
- Average representative distance of co-localization between cell type A and B: *sigma* =40.
- Change in co-localization between cell types between groups: *delta* = *sigma*/3.

For each simulation, cells in each image were simulated by first generating cells from cell type A using a Poisson point process model with expected count equal to a random value from *cellCount*. The density of cell type A is then calculated using a disc kernel, where the size of the disc for each subject is drawn from a Poisson with expected value *sigma* or *sigma + delta* depending on which group that subject belongs to. In the *difference* simulations the average co-localization is equal to *sigma* in the first group and *sigma* + *delta* in the second group. In the *null* simulations the average co-localization in the first group of subjects is the same as that in the second group of subjects. The cells from cell type B are then generated using a Poisson point process model with expected count equal to another random value from *cellCount* multiplied by the density of cell type A. When applying spicyR to the simulations, we apply Equation (3) across a range of radii (*r’* *=* 10, 20, …, 100) to obtain the co-localization statistic.

To evaluate the performance of spicyR, the *P*-value distributions from the tests on the *null* simulations were compared to the *difference* simulations. The percentage of *P*-values from the *null* simulations less than a significant threshold will provide a false positive rate and the percentage of *P*-values from the *difference* simulations less than a significance threshold will provide a true positive rate.

The simulations were first applied to identify if the use of the weighting regime as described above improved the sensitivity of spicyR. Next, simulations were performed to explore the sensitivity of spicyR to different average co-localization distances. This was achieved by repeating the above simulations, while also varying *sigma* from 10 up to 100. spicyR is then applied by applying Equation (3) across a range of radii (*r’* *=* 10, 20, …, 100), or by taking the difference between discrete *L* curve values for each *r* from 10 up to 100. Finally, the simulations were used to evaluate how spicyR performs for different cell counts with *cellCount* multiplied by a factor of 1, 5, 10 and 20.

### 2.5 Application to diabetes IMC data in Damond *et al.* (2019)

As an implemented example of the framework, we apply spicyR to the data presented by Damond *et al.* This study aimed to identify the spatial distribution of markers and cells in pancreatic islets, comparing three different stages of type 1 diabetes mellitus (T1DM) progression: non-diabetic, onset diabetes and long-duration diabetes, with four subjects per group. The single-cell image data were downloaded from Mendeley (Version 2: https://data.mendeley.com/datasets/cydmwsfztj/2). This dataset consists of images of pancreatic islet cells across the three disease stages, obtained via IMC with 37 markers. The number of images per subject ranged from 64 to 81 (Total= 845; Non-diabetic = 274; Onset = 290; Long-duration = 281). Additionally, the dataset contained spreadsheets providing relevant patient information, as well as the cell coordinates, marker expression and cell classification.

Here, we aimed to apply spicyR to identify significant differential co-localizations across the different disease stages. The *X* and *Y* coordinates of each cell, which image the cell belonged to, and the cell type were obtained, alongside the patient ID and diabetes disease stage for each image. spicyR was then applied to the data, with patient ID being treated as the random effect and the disease stage (non-diabetic, onset and long-duration diabetes) being treated as the fixed effect. The co-localization statistic was obtained by applying Equation (3) across a range of radii (*r’* *=* 10, 20, …, 100). The cell types studied here are the endocrine cell subsets (alpha, beta, gamma and delta) and the immune cell subsets [T helper (Th), T cytotoxic (Tc), neutrophils and macrophages].

### 2.6 Application to other datasets

The IMC dataset from Damond *et al.* has many images per patient and a categorical outcome with three categories. To demonstrate that spicyR can be applied to datasets with one image per patient, fit models which include covariates and binary outcomes and demonstrate variation in computational performance we applied spicyR to a subset of a dataset consisting of breast cancer patients assayed with IMC (Jackson *et al.*, 2020) available in the imcdatasets R package, and colorectal cancer patients assayed with CODEX (Schürch *et al.*, 2020).

## 3 Results

Here, we present spicyR, a framework for identifying changes in spatial association between pairs of cell types that could be associated with images from different clinical or experimental groups (Fig. 1). As input, spicyR requires images that have undergone single-cell segmentation and cell-type classification. In addition to calculating measures of co-localization between pairs of cell types [Fig. 2(A–C)], we provide functionality for empirically estimating the variability of the spatial associations between cell types within an image and for including multiple images per subject in the models.

We observed across multiple datasets (Fig. 2D and Supplementary Fig. S1) that the variability of the measure of spatial association between a pair of cell types, *u*, decrease as the number of the cells in an image increase. Quantifying the relationship between the variability of *u* and the number of cells in an image, provides an opportunity to propagate this information in the model fitting process. We model the relationship between *u* and cell count (Fig. 2D) by fitting a shape constrained GAM to the square of *u* as a function of the counts in both cell types. The inverse of the values obtained from this fitted curve are then used as weights (Fig. 2E) when testing for association between the co-localization of a pair of cell types and an outcome, with lower weights being applied to images with lower cell counts for a given cell type.

**Fig. 3.**
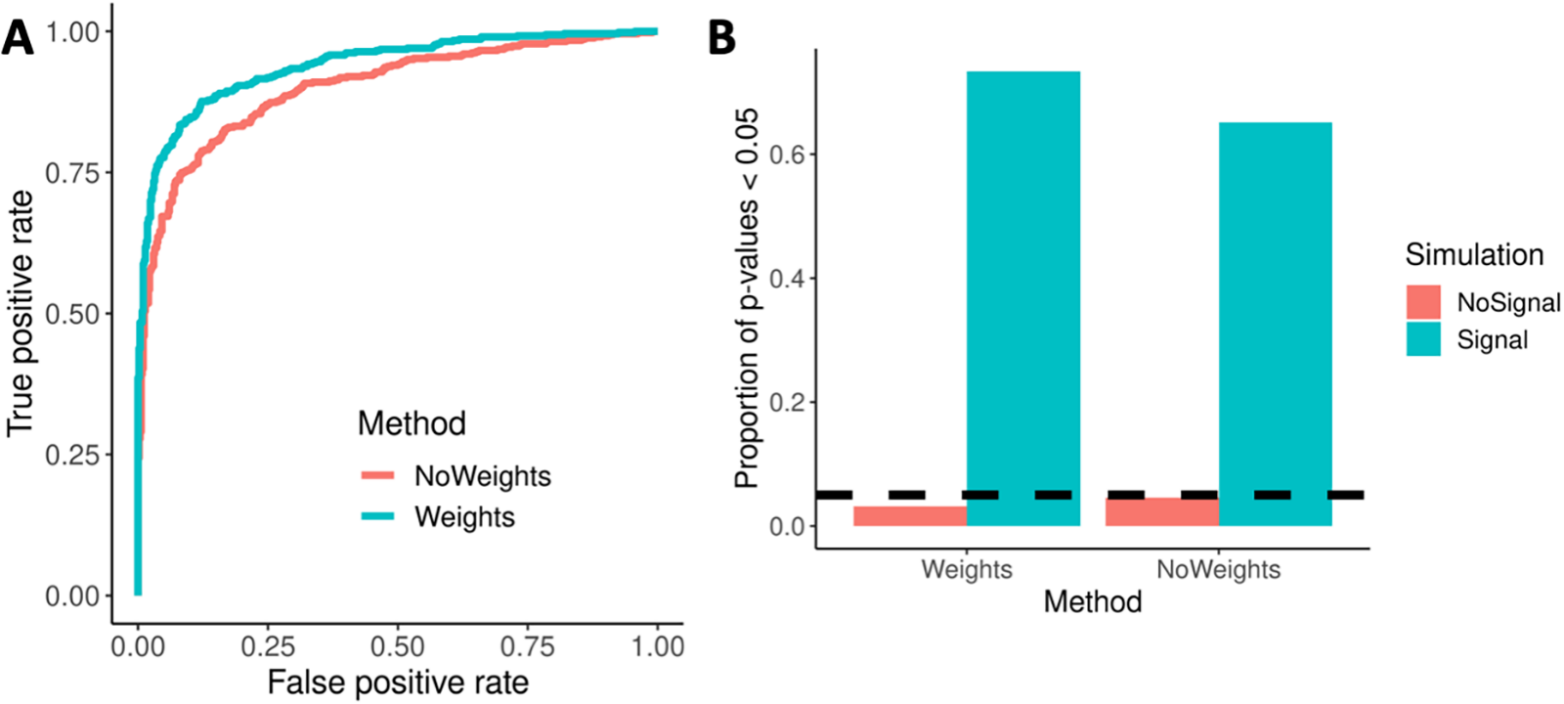
Simulations demonstrating the performance of the spicyR framework. (**A**) ROC curves obtained from simulations (AUC: no weights = 0.901; weights = 0.940). (**B**) Bar plots of the percentage of null simulations (false positives) and simulations with changes in co-localization (true positives) that were significant at a *P* =0.05 level when spicyR was applied

Simulated images were generated using the spatstat package to assess the performance of spicyR. First, we examined the benefits of including image weights in spicyR where we observed a distinct difference in the area under the receiver operating characteristic curves (AUC) with tests that included weights performing better than the non-weighted tests (AUC values: no weights =0.901; weights =0.940) (Fig. 3A and B). Next, we explore the role of cell counts on the performance of spicyR where the performance benefit from using weights was retained as the average number of cells in an image increased (Supplementary Fig. S3A and B). We further assessed how cell count influences computation time (Supplementary Fig. S3C) with a simulated image with 1000 cells taking ∼1 s to calculate pairwise co-localizations. To complement this, we have included the computational times of three publicly available datasets in Supplementary Table S1. Finally, we examined the sensitivity of spicyR to the choice of radii for calculating the co-localization score. In all simulations, the performance of spicyR was superior when the choice of radius used to quantify co-localization was close to the distance, which was used to generate the cell-type relationships (Supplementary Fig. S4A). However, running spicyR with a range of radii had the highest performance on average across all simulations (Supplementary Fig. S4B). This indicates that when there is uncertainty around the distance which changes in co-localization are occurring, as in most cases, choosing a range of radii might be an effective strategy for detecting those changes. When the expected co-localization distance is known, the use of a single radius may be sufficient. Nevertheless, in most circumstances, it is recommended to use multiple radii to define the co-localization statistic.

Damond *et al.* aimed to identify spatial changes in marker and cell distribution in pancreatic islets of three T1DM disease groups: non-diabetic, onset diabetes and long-duration diabetes. A key finding of the study was that there was a temporal correlation between beta cell destruction, marked by marker loss and cell decreases, and an increased infiltration of Th and Tc cells in beta cell-rich pancreatic islets. Hence, Th and Tc cells were implicated in the destruction of beta cells characteristic of T1DM. We aimed to explore these results further, using spicyR to identify differential cell-type co-localizations between the non-diabetic group compared to the onset diabetes group. The cell types included were the endocrine cell subsets (alpha, beta, gamma and delta) and the immune cell subsets (Th, Tc, neutrophils and macrophages).

**Fig. 4.**
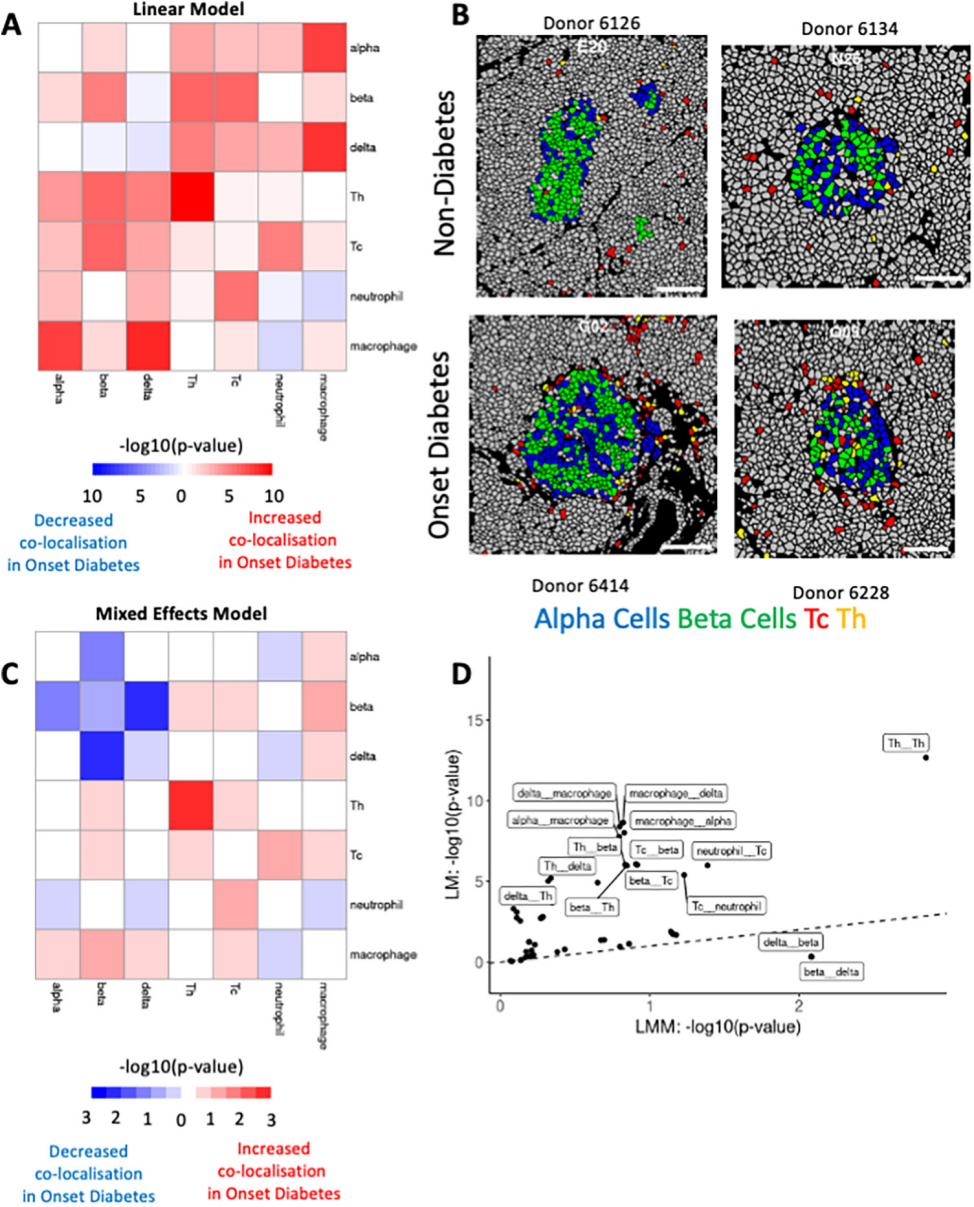
Application of spicyR to the Damond *et al.* (2019) type 1 diabetes IMC dataset. The spicyR framework was used to compare changes in cell-type co-localizations between the non-diabetic group and the onset diabetes group. (**A**) Heatmap showing the −log10 *P*-value from applying spicyR using a linear model. The *y*-axis represents cell type *i* (the ‘from’ cell type), and the *x*-axis represents cell type *j* (the ‘to’ cell type). Positive values represent increased co-localization in the onset diabetes group; negative values represent decreased co-localization in the onset diabetic group. (**B**) Masks showing representative images from the non-diabetes group and the onset diabetes group. Four cell types are highlighted: Alpha cells, Beta cells, Tc cells and Th cells. (**C**) Heatmap showing the −log10 *P*-value from applying spicyR using a mixed-effects model. The *y*-axis represents cell type *i* (the ‘from’ cell type), and the *x*-axis represents cell type *j* (the ‘to’ cell type). Positive values (red) represent increased co-localization in the onset diabetes group; negative values (blue) represent decreased co-localization in the onset diabetic group. (**D**) Scatterplot showing −log10 *P*-values from the application of spicyR with a linear model ignoring patient information and a linear mixed model including patient information

First, we applied spicyR by modelling cell-type co-localization with a simple linear model, which ignores patient information. Concordant with the findings in Damond *et al.*, we observed that Th and Tc cells showed increased spatial co-localization with islet cells in the diseased groups, specifically towards beta cells (Fig. 4A and B). The T cell co-localizations were more significant with beta cells (from beta to Tc: *P* = 9.49 × 0^−7^; from beta to Th: *P* = 1.06×10^−6^) compared to alpha cells (alpha to Tc: *P* = 1.91×10^−3^; alpha to Th: *P* = 1.55×10^−4^). This suggests a migration of Th and Tc cells towards beta cells (Fig. 4B), specifically during the early disease stages, reiterating the results presented by Damond *et al.* It was also found that there was increased co-localization of Th cells in onset diabetics (from Th to Th: *P* = 2.15 ×10^−13^).

Next, we repeated this analysis, implementing a linear mixed-effects model, which explicitly modelled islets as coming from different individuals. In contrast to the previous model, immune interactions with beta cells did not appear to be significant (from beta to Tc: *P* = 0.121 from beta to Th: *P* = 0.142). This difference in result is attributed to the strong increase in co-localization only being present in two of the four onset diabetes patients (Supplementary Fig. S5A and B). However, using the mixed-effects model there was a decrease in co-localization between delta cells and beta cells (from beta to delta: *P* = 0.008, Supplementary Table S2) suggesting a pattern to the way that beta cells degrade in the islet. The differences between the linear model and the linear mixed-effects model are highlighted in Figure 4D.

## 4 Discussion

Here, we have presented spicyR, a tool for identifying differential cell-type co-localizations across different groups. We have demonstrated its performance to identify cell co-localization changes through both simulated images, and with application to a type 1 diabetes IMC dataset. Simulations revealed that including weights that quantified the relationship between the number of cells in an image and the variability of a co-localization statistic increased the sensitivity and specificity of spicyR. Furthermore, when spicyR was applied to the diabetes dataset, the original results were reaffirmed and other key cell interactions present in diabetes progression were highlighted.

The spicyR package has many advantages compared to other differential co-localization strategies used in high parameter image analysis. The key advantage of the package is its ability to summarize changes in cell-type co-localizations across groups of images. If multiple images are obtained from multiple subjects, the mixed-effects model implemented allows variations within each subject to be modelled. By taking values of the *L* curve at multiple radii, we obtain a co-localization statistic that is easily interpretable and comparable across images. Furthermore, we implement a weighting scheme to account for variation in the co-localization statistic given the pairwise cell-type counts, which improves the predictive capabilities of spicyR. Finally, the package allows differential cell-type co-localizations to be identified across all pairwise cell types within the dataset, summarized in an interpretable heatmap. In this way, spicyR provides the framework for highlighting key cell–cell interactions that change across groups within a high parameter imaging dataset.

Despite the advantages discussed, there are several limitations to the implementation and evaluation of spicyR. Firstly, to benefit from the weighting regime, a moderate number of images are required to better model the relationship between cell count and co-localization score. Depending on the experimental approach, and the availability of biological samples, this may not be feasible. To address this, we recommend calculating weights using information from all pairwise comparisons. Secondly, if there is confounding between cell number and the degree of change in co-localization between cell types, the weighting strategy might obscure these differences. Thirdly, there are trade-offs to using small versus large radii when applying spicyR. Large radii may provide a better overall summary of cell-type co-localization within an image, but can have decreased sensitivity, particularly if the co-localization occurs strongly only over short distances. Hence, users should choose an appropriate distance or range of distances based on the biological questions being studied. Finally, while computational simulations were performed, it is difficult to validate spicyR within biological scenarios. There may be further scope for producing biologically relevant ground truths with which spicyR can be tested against, as well as more complicated simulation studies to elicit the effectiveness of the package.

It is important to acknowledge that spicyR should not be used in isolation to other analysis techniques, with the package being a key step in a broader analysis pipeline for high parameter imaging data. Firstly, spicyR is contingent on single-cell segmentation and cell-type classification (unsupervised or manually gated) being sufficiently accurate to elicit biologically relevant results. It is also complementary to other tests, such as testing for changes in cell-type composition and cell marker expression (Baharlou *et al.*, 2019), which are necessary for providing an overview of the biological process being studied. Furthermore, it is advised to explore images visually both before and after any spatial analysis to identify whether visual observations appear to be consistent with the results of spicyR. Tools, such as histoCAT (Schapiro *et al.*, 2017) and the Bioconductor package cytomapper (Eling *et al.*, 2020), provide useful exploratory tools for facilitating such exploratory data analysis. Ultimately, the package serves the role of highlighting changes in cell-type co-localization. This information can be crucial for synthesizing key biological insight from multiplexed imaging experiments. Overall, results from spicyR will complement observations obtained from other elements of an image analysis pipeline.

## Funding

This work was partly supported by the University of Sydney and an Australian Research Council Discovery Early Career Researcher Award [DE200100944] funded by the Australian Government. The funders had no role in the study design, data collection and analysis, decision to publish, or preparation of the manuscript.

*Conflict of Interest*: none declared.

## Data availability

The data underlying this article are available in https://github.com/nickcee/spicyRPaper.

## Supplementary Material

btac268_Supplementary_Data
