# Supplementary material for: spicyR: spatial analysis of in situ cytometry data in R
Source: Bioinformatics. 2022 Apr 19;38(11):3099–105. doi: 10.1093/bioinformatics/btac268 (PMC9326848; doi:10.1093/bioinformatics/btac268)
Supplement: btac268_Supplementary_Data [file btac268_supplementary_data.zip › Supplementary File.pdf]

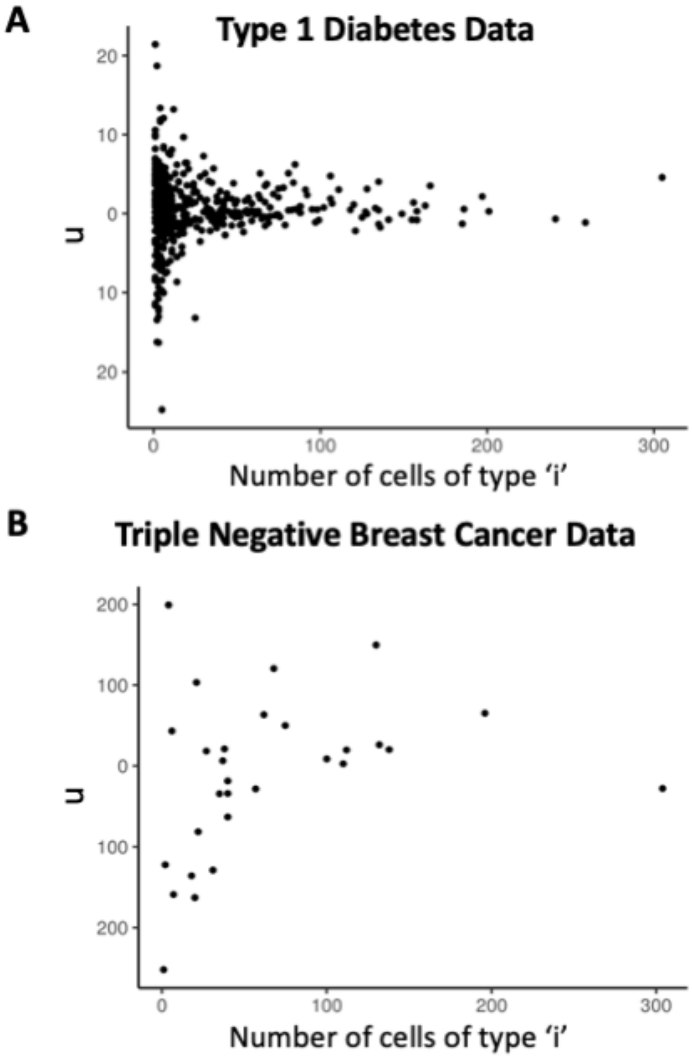

Figure S1. Relationship between the number of cells and variability of the quantification of localisation.

u as a function of the number of cells of type ‘i’ for each image. As the number of cell types is decreased, the number variation in the u statistic is increased. This is seen across three different datasets:

(A) Imaging mass cytometry images of type 1 diabetes samples from Damond et al. 2019

(B) Multiplexed ion beam imaging by time-of-flight (MIBI-TOF) images of triple negative breast cancer samples from Keren et al. 2018.

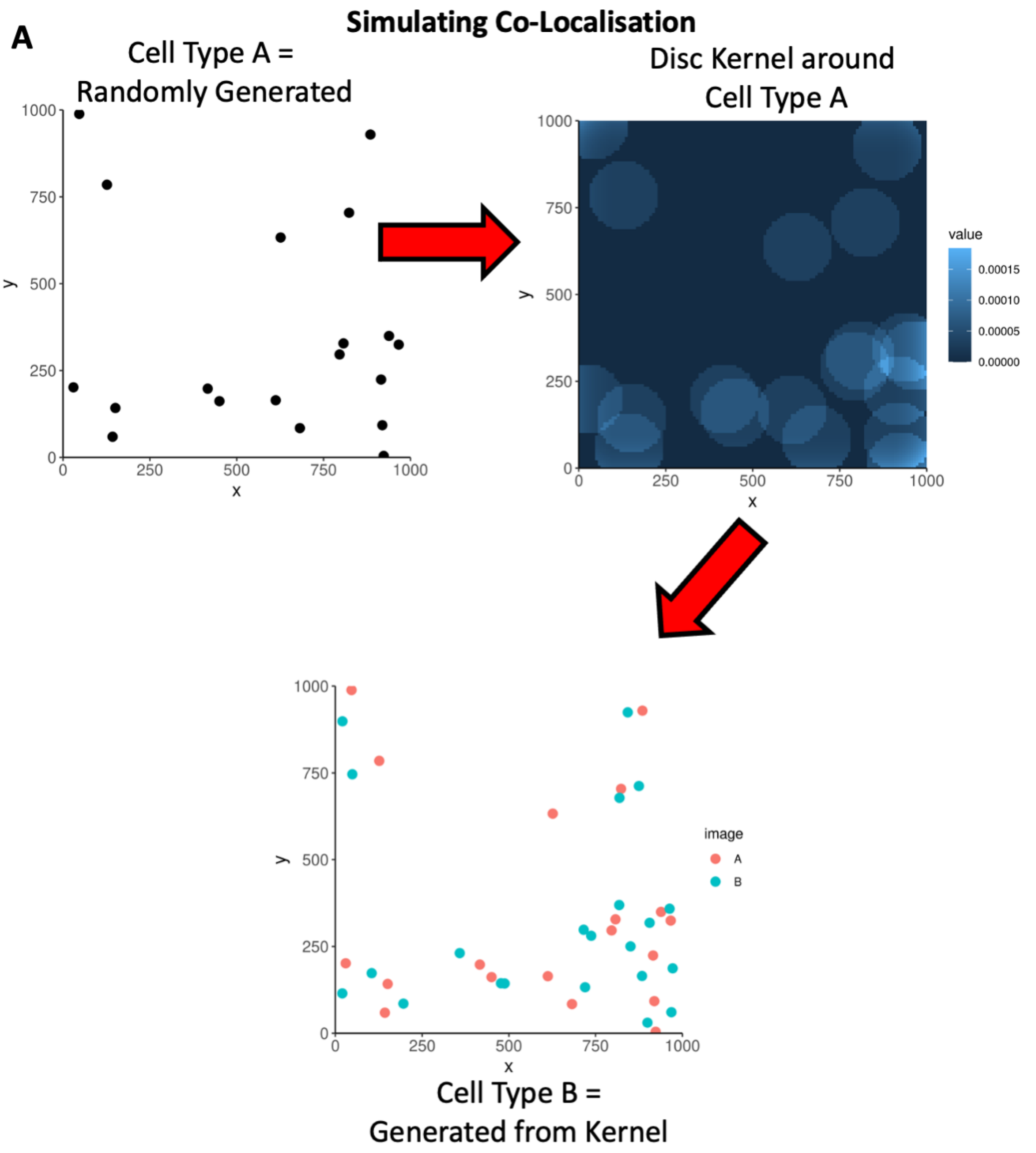
A
Simulating Co-Localisation
Cell Type A =
Randomly Generated
Disc Kernel around
Cell Type A
value
0.00015
0.00010
0.00005
0.00000
image
A
B
Cell Type B =
Generated from Kernel

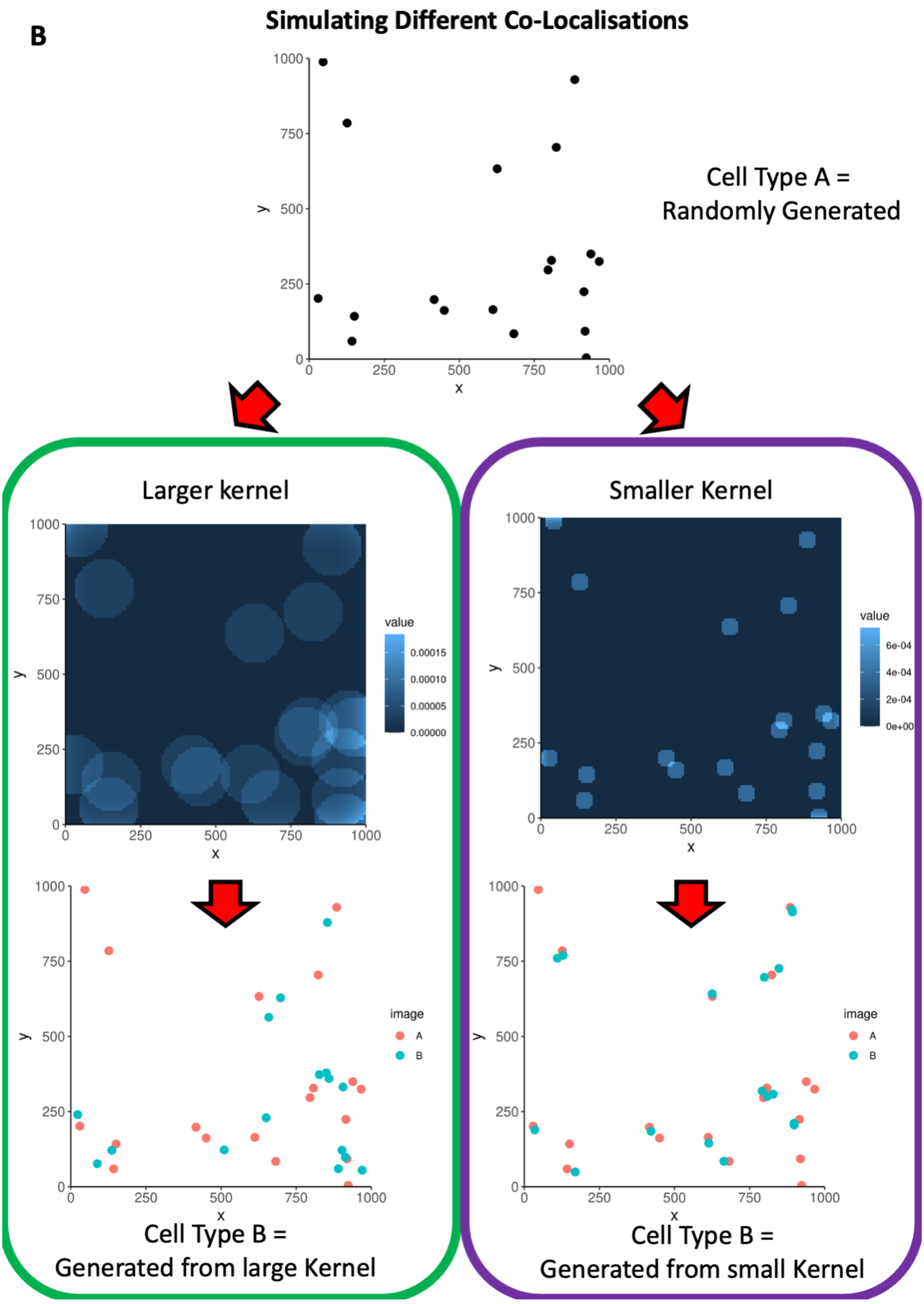

B
Simulating Different Co-Localisations
Cell Type A =
Randomly Generated
Larger kernel
value
Smaller Kernel
value
image
A
B
image
A
B
Cell Type B =
Generated from large Kernel
Cell Type B =
Generated from small Kernel

Figure S2. Summary of how simulations are performed

(A) Schematic of how co-localisation is simulated. Cells in cell type A is randomly generated with Poisson point process model. The density of cell type A is then calculated using a disc kernel, where the size of the disc is of a specified value. Cells from cell type B are then generated with a Poisson point process model within the kernel.

(B) Different levels of co-localisation are generated using different sized kernels.

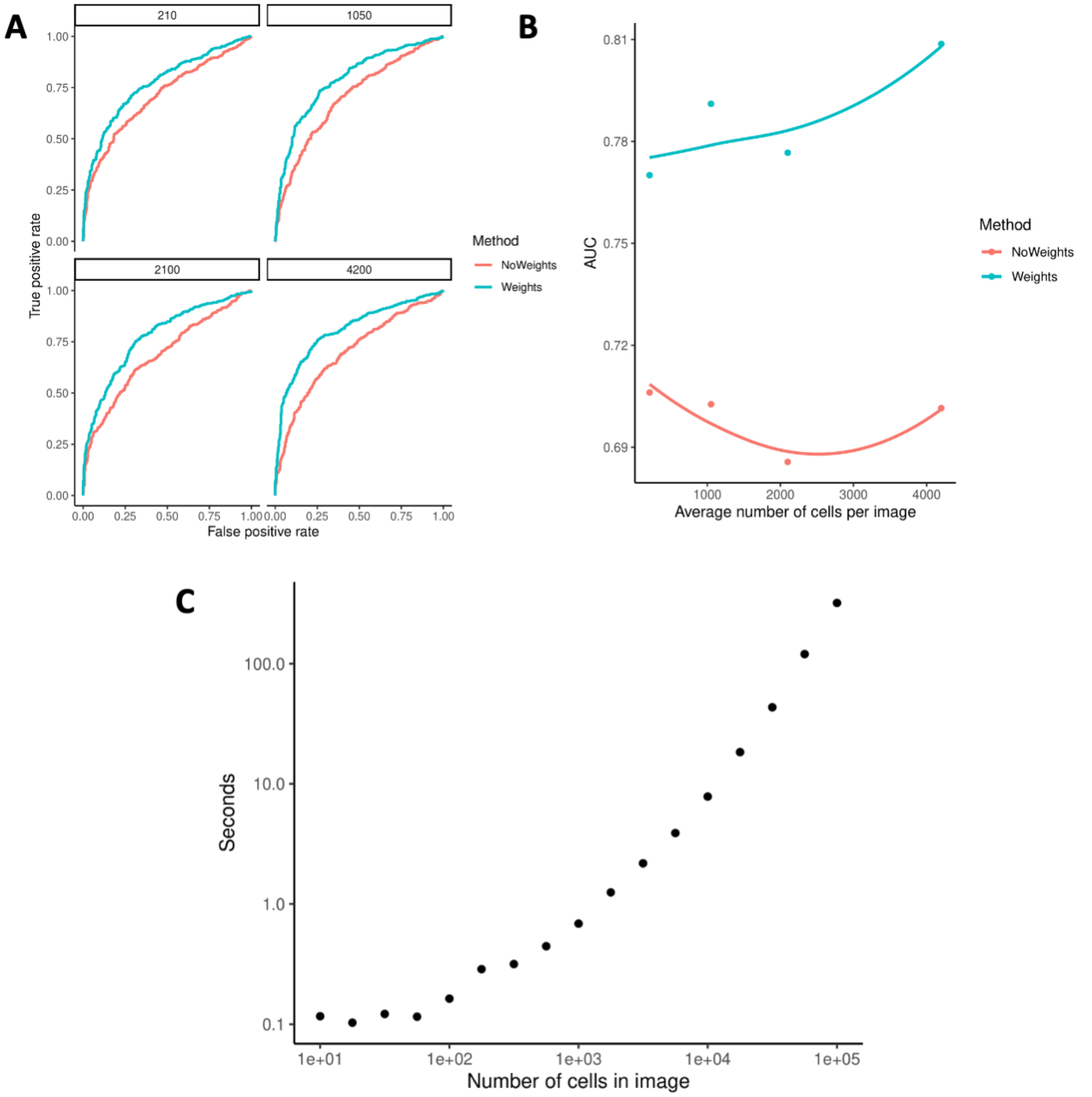

Figure S3. Simulations demonstrating the performance of the spicyR framework with varying cell counts.

Simulations were performed to demonstrate the performance of spicyR as the average number of cells of the two cell types being compared are increased. The simulation described in the Methods section is modified so that the number of cells in each image are multiplied by either 1, 5, 10 or 20 in respective simulations.

(A) The receiver operating characteristic curve for each simulation with the average for each simulation, with the average number of cells in each image list labelled above.

(B) Summary of the area under the curve of the receiver operating characteristic curve (AUC) for each simulation, as a function of average cell counts.

(C) Simulations were performed to calculate the time taken to calculate L-functions for images with increasing cell numbers.

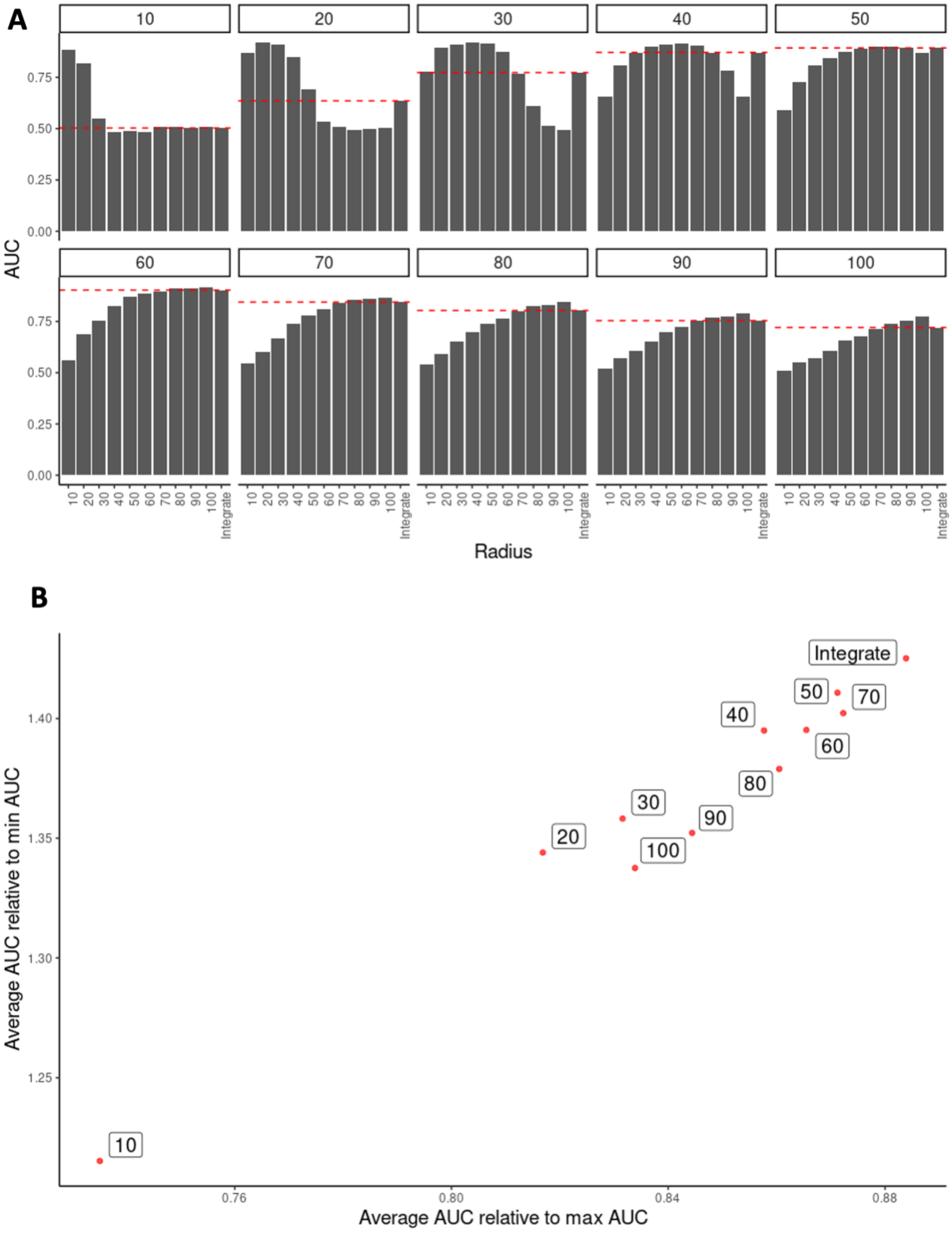
A
10
20
30
40
50
60
70
80
90
100
AUC
Radius
Integrate
0.00
0.25
0.50
0.75
B
Average AUC relative to min AUC
Average AUC relative to max AUC
Integrate
0.76
0.80
0.84
0.88
1.25
1.30
1.35
1.40

Figure S4. Simulations demonstrating the performance of the spicyR framework with varying degrees of co-localisation.

Simulations were performed to demonstrate the performance of spicyR as the co-localisation distance between cell types varies.The parameter lambda, which controls the degree of co-localisation of cell type A and cell type B, was varied from 10 to 100.

(A) AUC values for simulations with varying co-localisation distances, given by the heading. The x-axis represents the distance at which a co-localisation score was calculated, or if it was calculated through integrating (‘Integrate’). The red line signifies the AUC obtained when integrating to measure the co-localisation score.

(B) Values in (A) were averaged across all simulations give the average AUC relative to the best performing radius in each simulation (x-axis) and the average AUC relative to the worst performing radius in each simulation (y-axis).

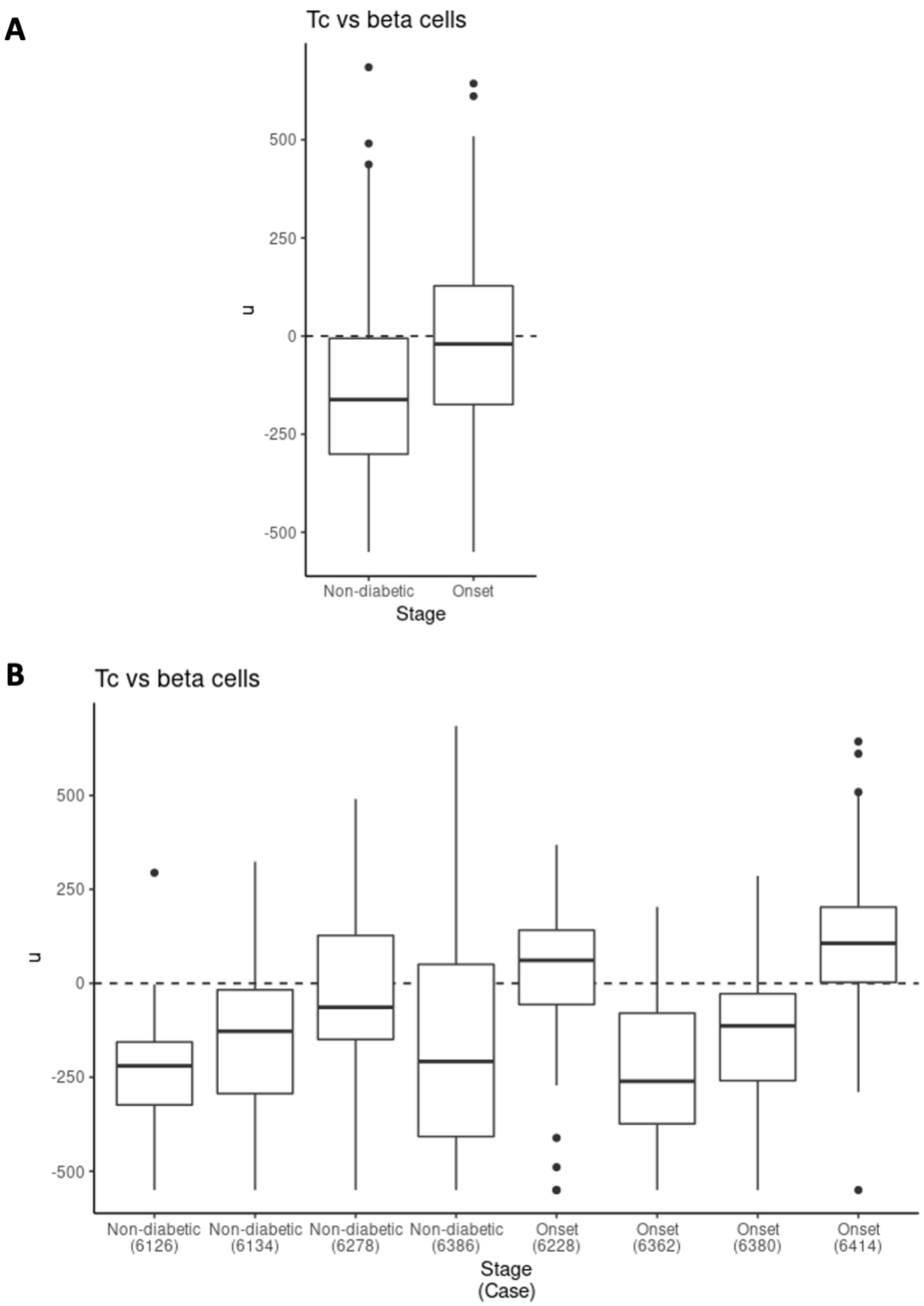

Figure S5. Cytotoxic T cells avoid beta cells in non-diabetic islets.
(A) Boxplots quantifying the level of co-localisation, u, of Tc and beta cells in islets from non-diabetic and onset diabetes patients.
(B) The islets are stratified by patient. The difference in co-localisation in (A) is primarily driven by two patients in (B).

Table S1. Time to run spicyR for different multiplex imaging datasets.
Calculating the L curves for each image is the most time-consuming component of spicyR. This relationship is observed when applying spicyR to three different datasets; a) diabetes patients assayed with IMC by Damond et al. (2019), b) breast cancer patients assayed with IMC by Jackson et al. (2020) and c) colorectal cancer patients assayed with CODEX by Schürch et al. (2020).

Dataset	Technology	Number of images	Average number of cells per image	Number of subjects	Number of cell types	Time with one core (minutes)	Time with eight cores (minutes)
Damond et al. (2019)	IMC	845	2100	12	16	63	13.5
Jackson et al. (2020)	IMC	100	2851	100	27	6.3	3.1
Schürch et al. (2020)	CODEX	140	1680	35	13	2.7	1.4

Table S2. Results from applying spicyR to compare Onset diabetes with Non-diabetes data.
Fitting a mixed effects model with spicyR identifies four pairwise relationships with a nominal p-value less than 0.05. Reported from the model are the *Intercept* (the co-localisation score for the Non-diabetics), the *Coefficient* (the change in co-localisation in Onset diabetes relative to Non-diabetics), the *p-value* for the coefficient of change, the *Adjusted p-value* accounting for multiple testing (FDR correction), the first cell type (*From*) compared to the second cell type (*To*).

Intercept	Coefficient	P-value	Adjusted P-value	From	To
-117.34	273.45	0.0014	0.061	Th	Th
590.63	-184.00	0.0083	0.14	Beta	Delta
590.42	-182.44	0.0084	0.14	Delta	Beta
-67.16	74.88	0.042	0.31	Neutrophil	Tc
