# Supplementary figures and images for: spicyR: spatial analysis of in situ cytometry data in R
Source: Bioinformatics. 2022 Apr 19;38(11):3099–105. doi: 10.1093/bioinformatics/btac268 (PMC9326848; doi:10.1093/bioinformatics/btac268)

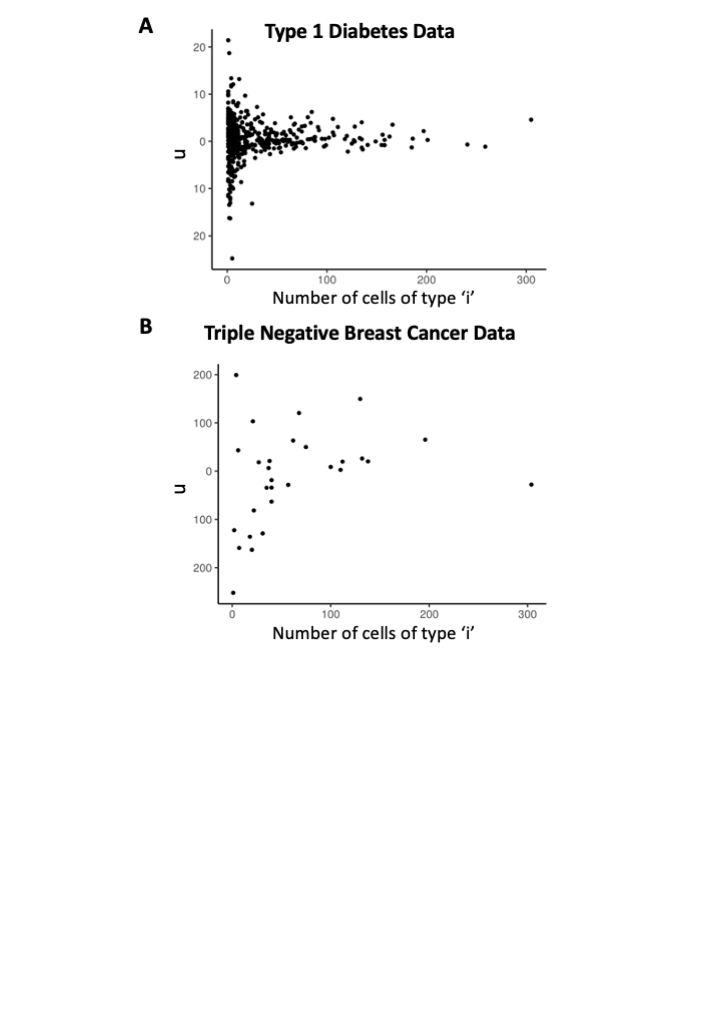

Supplement: btac268_Supplementary_Data [file btac268_supplementary_data.zip › FigS1.tiff]

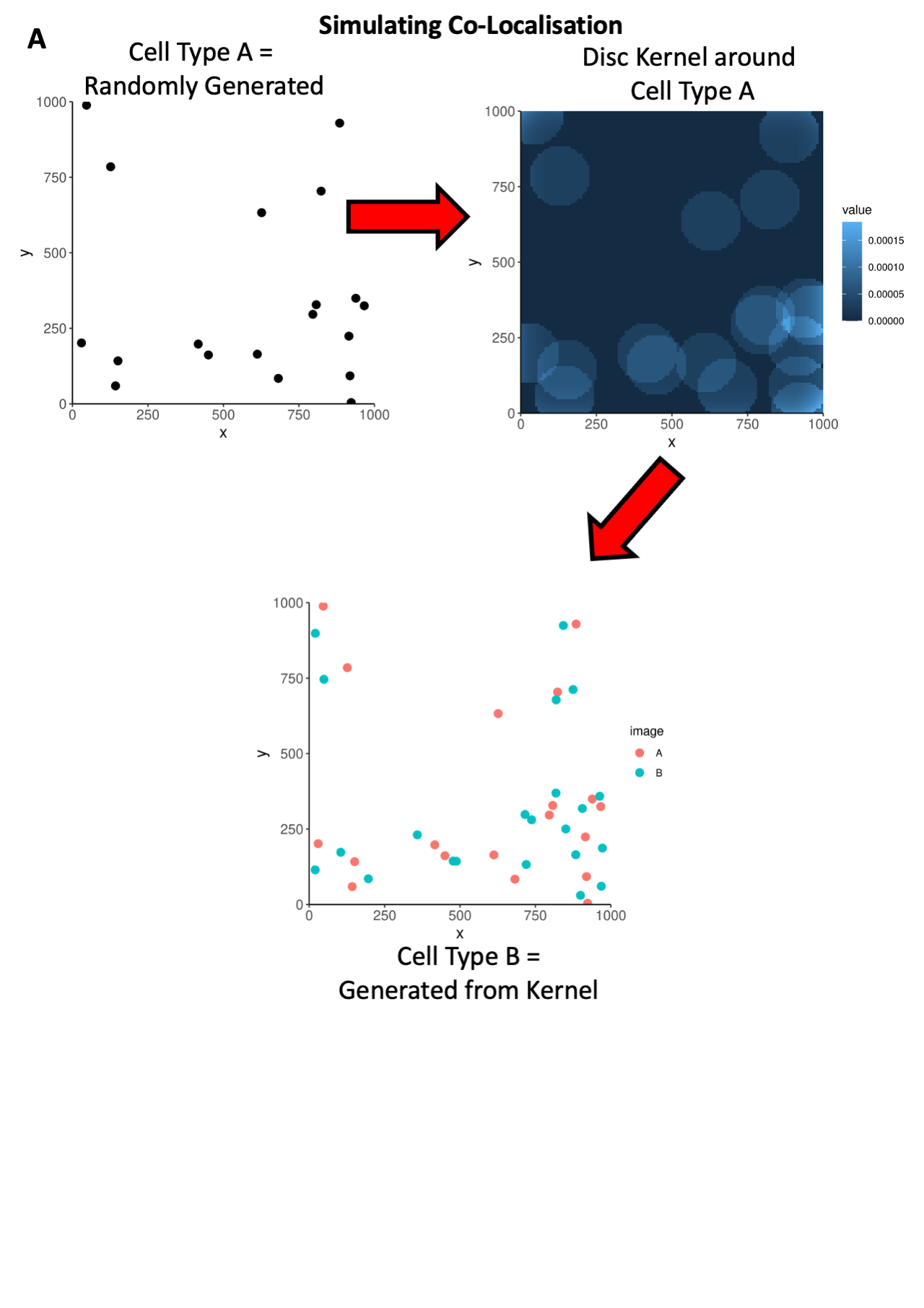

Supplement: btac268_Supplementary_Data [file btac268_supplementary_data.zip › FigS2A.tiff]

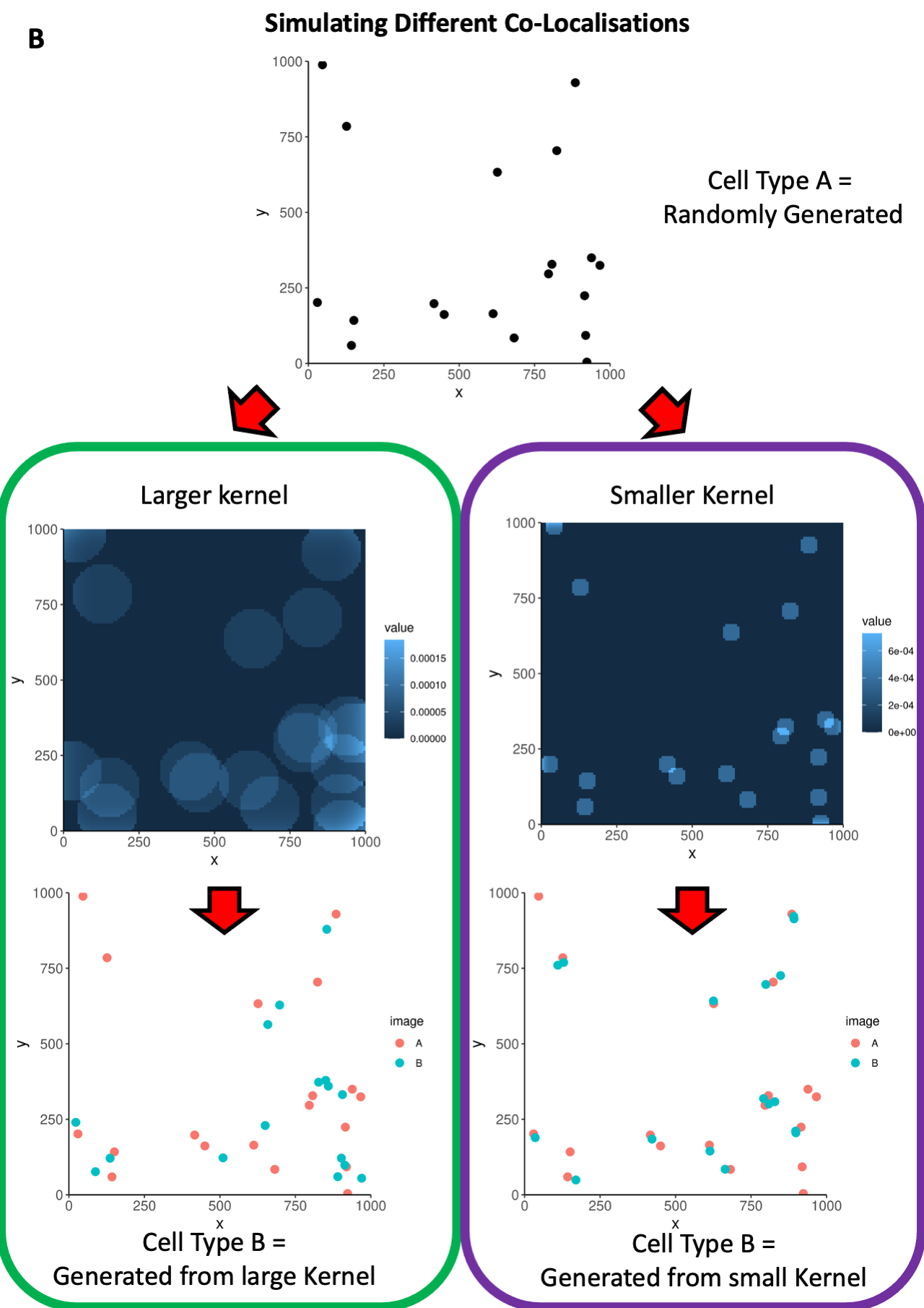

Supplement: btac268_Supplementary_Data [file btac268_supplementary_data.zip › FigS2B.tiff]

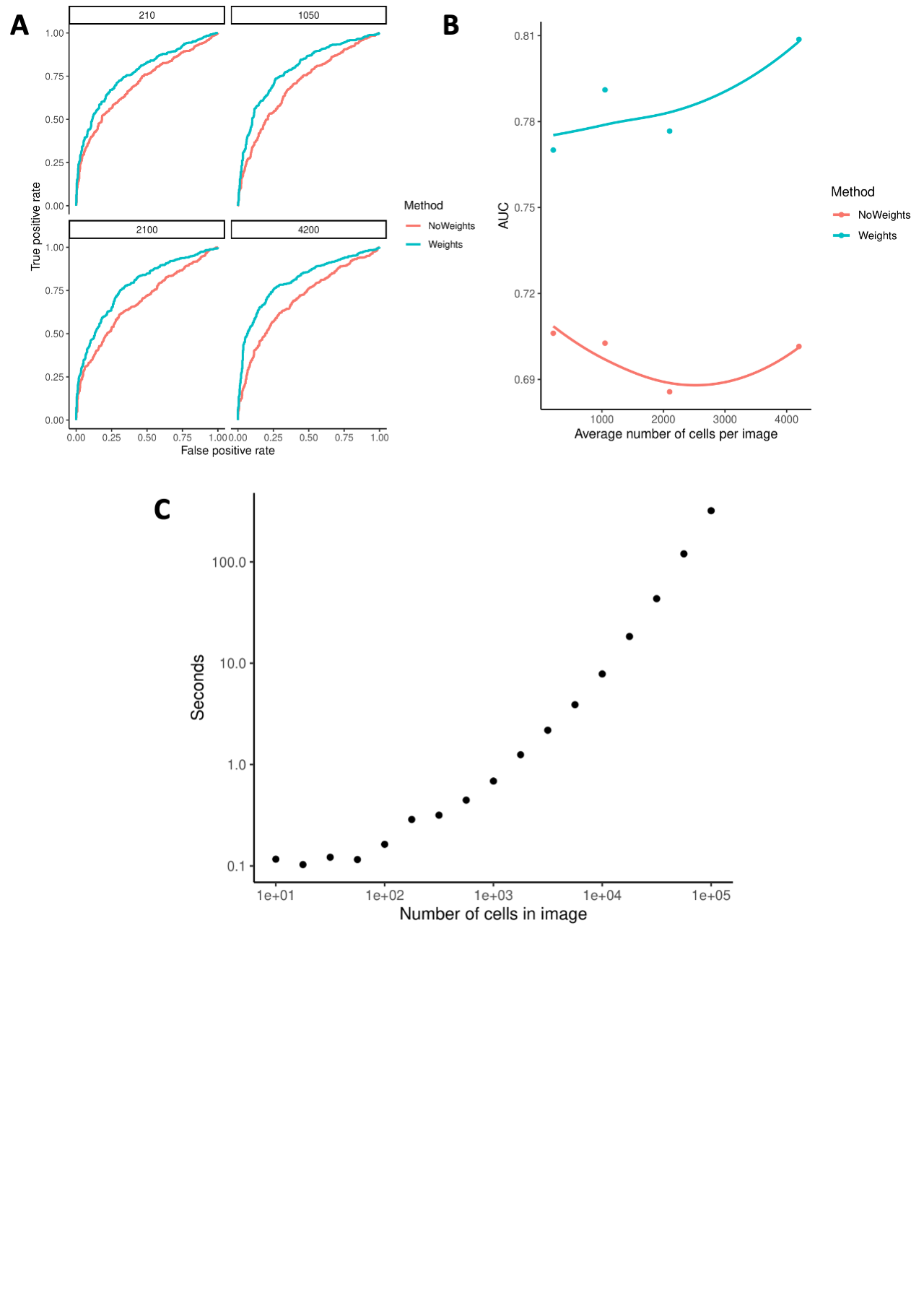

Supplement: btac268_Supplementary_Data [file btac268_supplementary_data.zip › FigS3.tiff]

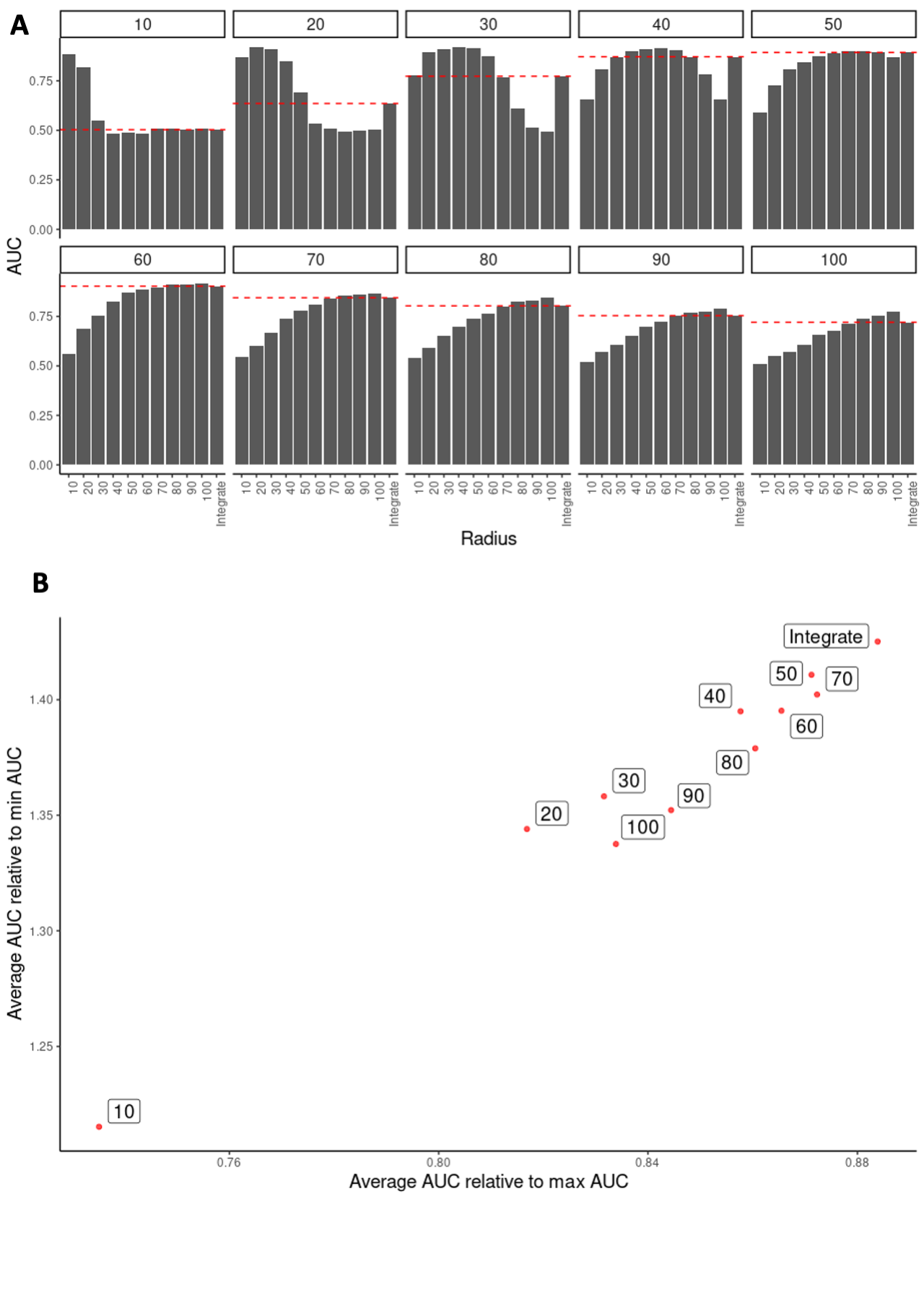

Supplement: btac268_Supplementary_Data [file btac268_supplementary_data.zip › FigS4.tiff]

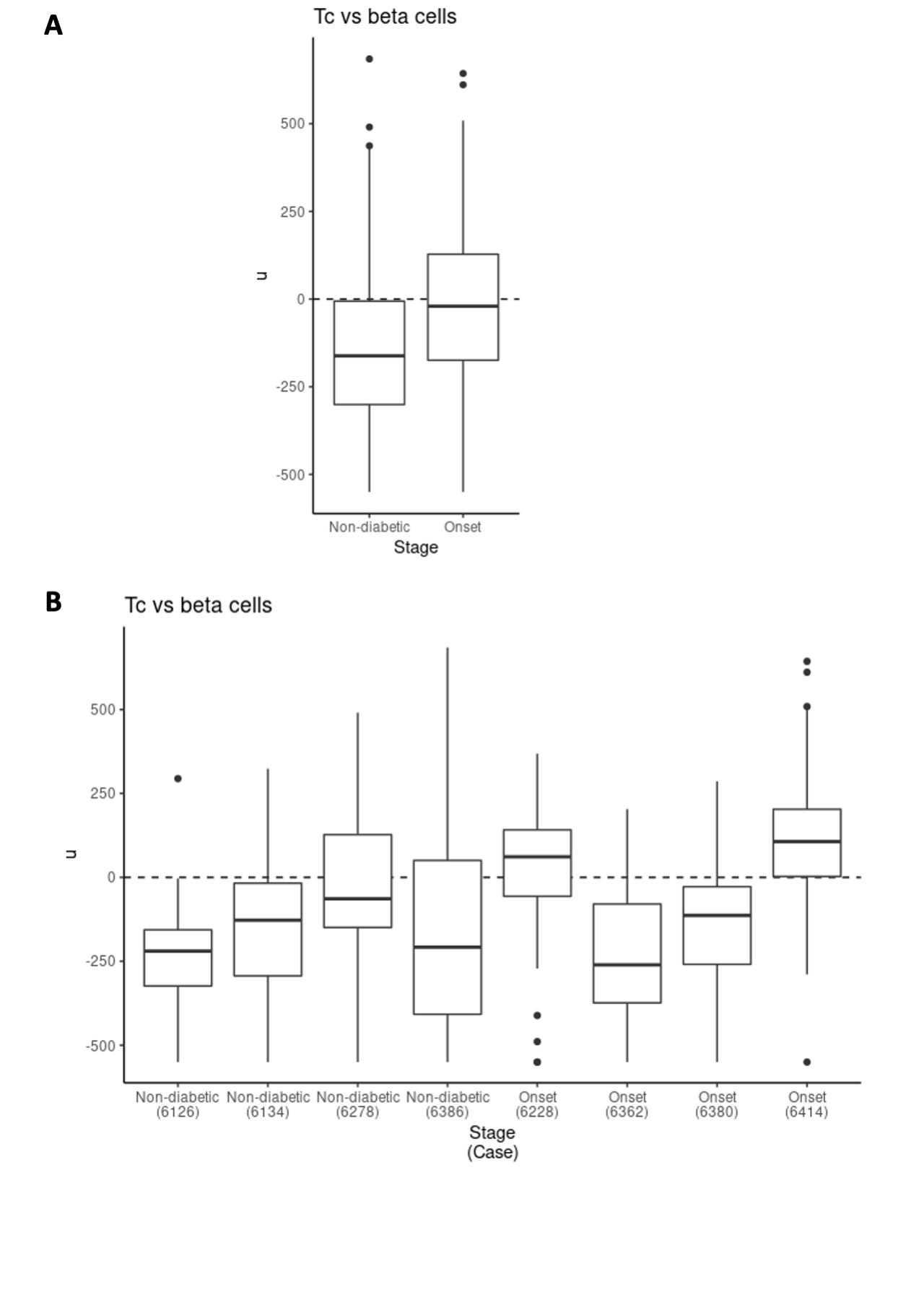

Supplement: btac268_Supplementary_Data [file btac268_supplementary_data.zip › FigS5.tiff]
